# Exploring the role of apolipoprotein ε4 in progressive myoclonic epilepsy type 1

**DOI:** 10.1002/epd2.70112

**Published:** 2025-10-03

**Authors:** Janina Gunnar, Yawu Liu, Henri Eronen, Tarja Joensuu, Marja Äikiä, Jelena Hyppönen, Katri Silvennoinen, Esa Mervaala, Juhana Hakumäki, Anna‐Elina Lehesjoki, Reetta Kälviäinen

**Affiliations:** ^1^ Institute of Clinical Medicine University of Eastern Finland Kuopio Finland; ^2^ Folkhälsan Research Center Helsinki Finland; ^3^ Medicum, University of Helsinki Helsinki Finland; ^4^ Kuopio Epilepsy Center, Kuopio University Hospital, Member of ERN EpiCARE Kuopio Finland; ^5^ Epilepsia Helsinki Helsinki University Hospital, Member of ERN EpiCare Helsinki Finland; ^6^ Imaging Center Kuopio University Hospital Kuopio Finland

**Keywords:** APOE, EPM1, gene, ULD, Unverricht‐Lundborg disease

## Abstract

**Objective:**

Progressive myoclonic epilepsy type 1 (EPM1) is a neurodegenerative disease caused by biallelic variants in the cystatin B (*CSTB*) gene. Despite a progressive course, phenotype severity varies among patients, even within families. We studied the potential role of *APOE* ε4 in modifying phenotypic diversity in EPM1, given its established association with neurodegeneration, particularly in Alzheimer's disease.

**Methods:**

*APOE* genotypes were determined for 65 genetically verified EPM1 patients homozygous for the CSTB expansion mutation. The Unified Myoclonus Rating Scale (UMRS), Quality of Life in Epilepsy Inventory‐31 questionnaire (QOLIE‐31), intellectual ability (WAIS‐R), clinical data, and quantitative neuroimaging data were compared between *APOE* ε4 carriers and noncarriers to assess potential correlations with EPM1 severity. Volumetric analysis was performed on MRI data, while diffusion tensor imaging (DTI) was analyzed using Tract‐Based Spatial Statistics (TBSS) and atlas‐based white matter (WM) tract region of interest (ROI) analysis.

**Results:**

The cohort included 20 ε4 carriers (16 ε3/ε4 and 4 ε4/ε4) and 45 ε4 noncarriers (36 ε3/ε3, 8 ε2/ε3, and 1 ε2/ε2). No significant differences were found in UMRS or disease duration. Carriers had better QOLIE‐31 scores in emotional well‐being (*p* = .047), energy/fatigue (*p* = .048), and medical effects (*p* = .024). In volumetric analysis, carriers exhibited greater preservation of bilateral hippocampal and amygdalar volumes but demonstrated more pronounced cortical thinning in the left lingual gyrus, right lateral occipital gyrus, and right posterior cingulate (*p* < .05). Carriers exhibited more widespread WM degeneration in DTI, characterized by reduced fractional anisotropy (FA) and increased mean diffusivity (MD).

**Significance:**

Despite greater white matter degeneration and reduced cortical thickness, *APOE* ε4 carriers exhibited preserved deep brain volumes and better self‐reported well‐being. This study highlights the complex interplay between genetic factors and neurodegenerative processes. Our future research aims to provide more natural history data of EPM1 and correlate long‐term phenotypic data with additional geno‐phenotypic analyses.

**Key points**

- Progressive myoclonic epilepsy type 1 (EPM1) is a genetic disorder causing myoclonus, seizures, and slow, progressive neurological decline.
- EPM1 patients exhibit high phenotypic variation, highlighting the need for identifying biomarkers to better understand disease mechanisms.
- Apolipoprotein E (*APOE*) ε4 is linked to other neurodegenerative diseases, suggesting a potential role in EPM1 progression.
- Patients with *APOE* ε4 had reduced cortical thickness and more severe WM loss but preserved hippocampal and amygdalar volumes.
- This study highlights the complex interplay between genetic factors and neurodegenerative processes.

## 1 INTRODUCTION

Progressive myoclonic epilepsy type 1 (EPM1), also known as Unverricht‐Lundborg disease (ULD), is a rare neurodegenerative disorder characterized by an age of onset from six to 16 years, stimulus‐sensitive myoclonus, and generalized tonic–clonic seizures.^1^ EPM1 is an autosomal recessive disease caused by biallelic variants in the cystatin B (*CSTB*) gene.^2^ It is the most common disorder among progressive myoclonic epilepsies, which collectively represent one of the most disabling forms of epilepsy.^3^ While EPM1 occurs worldwide, its prevalence (1:50 000) is highest in Finland.^4^ Although the course of the disease is unavoidably progressive, the phenotype of EPM1 can vary significantly even within the same family.^1^ Epileptic seizures are generally well controlled with medication, but myoclonus remains a disabling and treatment‐resistant symptom.^5^ Most patients lose the ability to mobilize without assistance due to worsening myoclonus and have difficulties with many activities of daily living, while verbal abilities and memory are less affected.^3^, ^6^

Structural brain imaging in individual EPM1 patients is typically within normal limits when visually assessed.^1^ Quantitative MRI studies have revealed regional bilateral gray matter volume loss in the motor cortex and thalamus, thinning of the sensorimotor, visual, and auditory cortices, and mild cerebellar atrophy, consistent with the motor symptoms and stimulus‐sensitive nature of the disease.^7^, ^8^, ^9^, ^10^ Diffusion tensor imaging (DTI) provides further insights into the microstructural changes underlying EPM1. Notably, reduced fractional anisotropy (FA) and increased mean diffusivity (MD) are commonly observed in the cerebellum, thalamus, and extensive white matter (WM) regions, reflecting widespread WM degradation and neuronal loss.^11^ Additionally, research using *Cstb*‐deficient mouse models has revealed notable WM degeneration, aligning with DTI findings in EPM1 patients.^12^ The findings suggest that WM degeneration may play a key role in the underlying pathology of EPM1, emphasizing the need for further investigation into both the genetic and structural factors shaping disease progression.

While previous studies have focused on defining the phenotype of EPM1, significant gaps remain in understanding the factors contributing to its phenotypic variation. In other neurodegenerative diseases, polymorphic apolipoprotein E (*APOE*) genotypes have been found to play a significant role. Individuals carrying the *APOE* ε4 allele have an increased risk of developing Alzheimer's disease^13^ and ischemic stroke,^14^, ^15^, ^16^, ^17^ and it may also define a more vulnerable subtype of Parkinson's disease.^18^ *APOE* ε4 also significantly increases the risk of hyperlipidemia and coronary artery disease in individuals with or without type 2 diabetes.^19^ Additionally, several other pathogenic associations have been identified.^20^ Neuroimaging studies in Alzheimer's disease have shown that ε4 carriers exhibit accelerated hippocampal and amygdalar atrophy, cortical thinning in memory‐related regions, and greater WM damage, particularly in the frontal, temporal, and cingulate regions of the brain.^21^, ^22^

This study aimed to explore the combined effects of EPM1 and the *APOE* ε4 allele on neuroimaging findings, with a particular focus on brain structure and microstructure, including volumetric changes, WM integrity, and diffusion metrics. Additionally, we examined how these imaging biomarkers correlate with disease severity, such as motor symptoms, cognitive function, and quality of life. We hypothesize that the presence of the *APOE* ε4 allele may accelerate neurodegeneration, exacerbating the atrophy and WM degeneration observed in EPM1, thereby leading to a faster progression of the disease.

## 2 MATERIALS AND METHODS

The data presented originates from a comprehensive, multidisciplinary study of the natural course of EPM1 in the Epilepsy Center at Kuopio University Hospital. This nationwide study includes genetically verified EPM1 patients in Finland who are over 12 years of age. Ethical approval for the study was granted by the Ethics Committee of Kuopio University Hospital (KUH), and written informed consent was obtained from all participants in accordance with the Declaration of Helsinki.

A total of 65 genetically verified EPM1 patients, all homozygous for the expansion mutation in the *CSTB* gene, were included in the study. Two *APOE* variants, rs7412 and rs429358 defining the alleles *ε2*, *ε3*, and *ε4*,^23^ were genotyped by bi‐directional Sanger sequencing (ABI BigDye 3.1, Applied Biosystems) on an ABI3730xl DNA Analyzer. Primers were designed with Primer‐BLAST, and sequences were analyzed using Sequencher v.5.3 (Gene Codes Corporation). All patients were treated with antiseizure and antimyoclonic medication, as well as routine clinical management for EPM1 in accordance with national epilepsy care guidelines at the time of participation. The clinical and neuropsychological assessment of the patients has been described earlier in detail.^24^ The cross‐sectional data collected included Unified Myoclonus Rating Scale (UMRS) scores,^25^ the Quality of Life in Epilepsy Inventory‐31 (QOLIE‐31) questionnaire,^26^ Verbal and Performance Intelligence Quotients (VIQ and PIQ, respectively) assessed with six subtests of the Wechsler Adult Intelligence Scale Revised (WAIS‐R),^27^ and MRI. UMRS scores included action myoclonus (AM), negative myoclonus, negative myoclonus severity, stimulus sensitivity (SS), and functional tests (FT). Clinical disease severity is assessed using the AM score within the UMRS, with higher scores indicating more severe myoclonus.^24^ For measures other than the UMRS, higher scores reflect better performance.

### 2.1 MRI protocol

All participants underwent MRI using a 1.5 T Siemens Avanto scanner (Siemens, Erlangen, Germany) with the following imaging protocol: T2‐weighted spin‐echo sequence, fluid‐attenuated inversion recovery (FLAIR) sequence, and T1‐weighted 3D magnetization‐prepared rapid acquisition gradient‐echo (MPRAGE) sequence. The imaging parameters for the MPRAGE sequence were as follows: repetition time (TR), 1980 ms; echo time (TE), 3.09 ms; flip angle, 15°; matrix, 256 × 256; and voxel size, 1.0 × 1.0 × 1.0 mm.

DTI was performed for 52 of the 65 EPM1 patients, including 16 *APOE* ε4 carriers (mean age: 30 ± 11 years, disease duration: 21 ± 10 years) and 36 noncarriers (mean age: 35 ± 12 years, disease duration: 24 ± 10 years). The imaging protocol used a gradient‐echo single‐shot echo‐planar imaging sequence with the following parameters: TR, 9800–10,100 ms; TE, 12300–12,500 ms; matrix, 128 × 128; field of view (FOV), 256 × 256 mm^2^; section thickness, 2 mm; and diffusion gradients (*b*‐values: 0 and 1000 s/mm^2^) applied in 30 directions.

All MRI images were visually inspected by an experienced neuroradiologist (Y.L.) to ensure image quality before further analysis.

### 2.2 Volumetric analysis

We performed voxel‐based morphometry (VBM) using T1‐weighted images (T1WI) within the Computational Anatomy Toolbox 12 (CAT12), integrated into the Statistical Parametric Mapping (SPM12) framework, to assess gray matter volume differences. The analysis followed standard procedures: (1) gray matter maps were normalized to the MNI152 space using nonlinear transformations; (2) images were modulated to preserve total gray matter volume during spatial normalization, ensuring accurate volumetric comparisons; and (3) normalized gray matter maps were smoothed with an isotropic Gaussian kernel (e.g., 8 mm full‐width at half maximum (FWHM)) to enhance the signal‐to‐noise ratio and account for interindividual variability.

To corroborate the VBM findings, we employed the standard FreeSurfer v7.0.4 pipeline to extract regional cortical thickness and volume metrics from each participant. Cortical measurements were obtained using the Desikan‐Killiany atlas for parcellation, while subcortical segmentations were derived from the Aparc + ASEG atlas.

### 2.3 Diffusion tensor imaging analysis

WM integrity was assessed using Tract‐Based Spatial Statistics (TBSS), a voxel‐wise statistical method for analyzing DTI data. The analysis was conducted using the FMRIB Software Library (FSL), following its standard pipeline: FA maps were aligned to a common template and projected onto a mean FA skeleton, representing the center of all tracts. The FA transformation matrix was then applied to MD maps.

To examine tract‐specific WM integrity, FA and MD values were extracted from the JHU ICBM‐DTI‐81 atlas and mapped to the corresponding TBSS skeletons.^28^

### 2.4 Statistical analysis

#### 2.4.1 Demographic and clinical comparisons

Independent *t*‐tests were used to compare continuous variables between groups. For categorical variables, Chi‐square tests of Independence were applied, with Fisher's exact test used for 2 × 2 tables and the Fisher–Freeman–Halton exact test used for larger contingency tables.

#### 2.4.2 VBM analysis

Group comparisons of gray matter volume were performed using general linear models in SPM12, controlling for total intracranial volume and disease duration as covariates. Results were corrected for multiple comparisons using the false discovery rate (FDR) approach, with a cluster extent threshold of 100 voxels.

#### 2.4.3 TBSS analysis

Statistical comparisons of FA and MD values were conducted using nonparametric permutation testing (5000 iterations) in *Randomize* (FSL). Disease duration was included as a covariate, and Threshold‐Free Cluster Enhancement was applied to improve sensitivity to spatially contiguous clusters while avoiding arbitrary thresholds. Family‐wise error (FWE) rate correction was applied, with statistical significance set at *p* < .05.

#### 2.4.4 Atlas‐based comparisons

Regional differences in cortical volume, cortical thickness, and DTI metrics were analyzed using MANCOVA with Bonferroni correction. Statistical significance was set at *p* < .05. Disease duration was controlled as a covariate, and total intracranial volume (TIV) was included in the analysis of volume metrics to account for individual differences in brain size.

## 3 RESULTS

### 3.1 Demographic and clinical scores

The cohort of 65 EPM1 patients included 20 ε4 carriers, of whom 16 were ε3/ε4 (24.6%) and 4 were ε4/ε4 (6.2%). The remaining 45 participants were ε4 noncarriers, comprising 36 ε3/ε3 (55.4%), 8 ε2/ε3 (12.3%), and 1 ε2/ε2 (1.5%) (Table 1).

**TABLE 1:** Demographics and clinical characteristics of *APOE* ε4 carriers and noncarriers of the EPM1 patients in the volumetric analysis^a^.

	Carriers 20	Noncarriers 45	*p*‐values
Age (years)	31 ± 11	35 ± 12	.249
Gender (female/male)	9/11	22/23	.492
Disease duration	21 ± 10	25 ± 10	.225
UMRS			
SS	2 ± 1	2 ± 2	.364
FT	9 ± 6	11 ± 8	.326
AM	40 ± 28	50 ± 30	.214
Negative myoclonus	0.4 ± 0.5	0.4 ± 0.5	.685
Negative myoclonus severity	0.6 ± 0.8	0.7 ± 0.9	.634
Global disability	2 ± 1	2 ± 1	.287
Walking ability^b^			.905
Independent	4/11	9/32	.709
Occasionally uses wheelchair	2/11	9/32	.698
Wheelchair‐bound	5/11	14/32	1.000
VIQ	89 ± 13	84 ± 15	.170
PIQ	78 ± 15	74 ± 14	.316
QOLIE‐31			
Seizure worry	75 ± 27 (20)	71 ± 23 (43)	.497
Overall quality of life	65 ± 17 (20)	62 ± 17 (43)	.499
Emotional well‐being	75 ± 14 (19)	66 ± 17 (43)	.047
Energy/fatigue	63 ± 16 (19)	53 ± 19 (43)	.048
Cognitive function	69 ± 27 (20)	60 ± 20 (43)	.133
Medical effects	71 ± 18 (19)	56 ± 27 (43)	.024
Social functioning	60 ± 28 (20)	52 ± 26 (43)	.288
Overall score	68 ± 18 (19)	59 ± 15 (43)	.062
Health score	64 ± 24 (18)	57 ± 20 (41)	.289

Abbreviations: AM, myoclonus with action; FT, functional tests; QOLIE‐31, the Quality of Life in Epilepsy Inventory‐31; SS, stimulus sensitivity; UMRS, Unified Myoclonus Rating Scale.

^a^ Continuous variables are expressed as mean ± standard deviation (SD), and categorical variables as *n*/*N* (number of cases/total sample size) unless otherwise specified. This format applies to Tables 1 and 2.

^b^ Only for AM scores ≥30.

The carriers and noncarriers did not differ in age, gender, disease duration, or clinical measures, including SS, FT, and AM scores, as well as negative myoclonus, VIQ, and PIQ. There was also no significant difference in walking ability between carriers and noncarriers with UMRS AM of 30 or higher. However, carriers had significantly higher QOLIE‐31 emotional well‐being scores (75 ± 14 vs. 66 ± 17, *p* = .047), energy/fatigue scores (63 ± 16 vs. 53 ± 19, *p* = .048), and medical effects scores (71 ± 18 vs. 56 ± 27, *p* = .024) than noncarriers. Other QOLIE‐31 subdomains, including seizure worry, overall quality of life, cognitive function, social function, and health score, did not show significant differences (Tables 1 and 2).

**TABLE 2:** Demographics and clinical scores of *APOE* ε4 carriers and noncarriers of the EPM1 patients in the DTI analysis.

	Carriers 16	Noncarriers 36	*p*‐values
Age (years)	30 ± 11	35 ± 12	.220
Gender (Female/male)	8/8	19/17	.545
Disease duration	21 ± 10	24 ± 10	.236
UMRS			
SS	1 ± 1	2 ± 2	.349
FT	9 ± 6	11 ± 8	.406
AM	41 ± 28	49 ± 30	.382
Negative myoclonus	0.3 ± 0.5	0.4 ± 0.5	.639
Negative myoclonus severity	0.6 ± 0.9	0.6 ± 0.9	.923
Global disability	2 ± 1	2 ± 1	.503
QOLIE‐31			
Seizure worry	72 ± 28 (16)	68 ± 23 (34)	.604
Overall quality of life	64 ± 18 (16)	63 ± 17 (34)	.832
Emotional well‐being	75 ± 15 (16)	65 ± 18 (34)	.059
Energy/fatigue	62 ± 16 (16)	52 ± 19 (34)	.069
Cognitive function	68 ± 29 (16)	59 ± 21 (34)	.201
Medical effects	71 ± 17 (15)	51 ± 26 (34)	.010
Social functioning	58 ± 28 (16)	52 ± 27 (34)	.478
Overall score	67 ± 18 (16)	59 ± 16 (34)	.104
Health score	61 ± 26 (16)	56 ± 21 (34)	.540

Abbreviations: AM, myoclonus with action; FT, functional tests; QOLIE‐31, the Quality of Life in Epilepsy Inventory‐31; SS, stimulus sensitivity; UMRS, Unified Myoclonus Rating Scale.

### 3.2 Volumetric analysis

**FIGURE 1:**
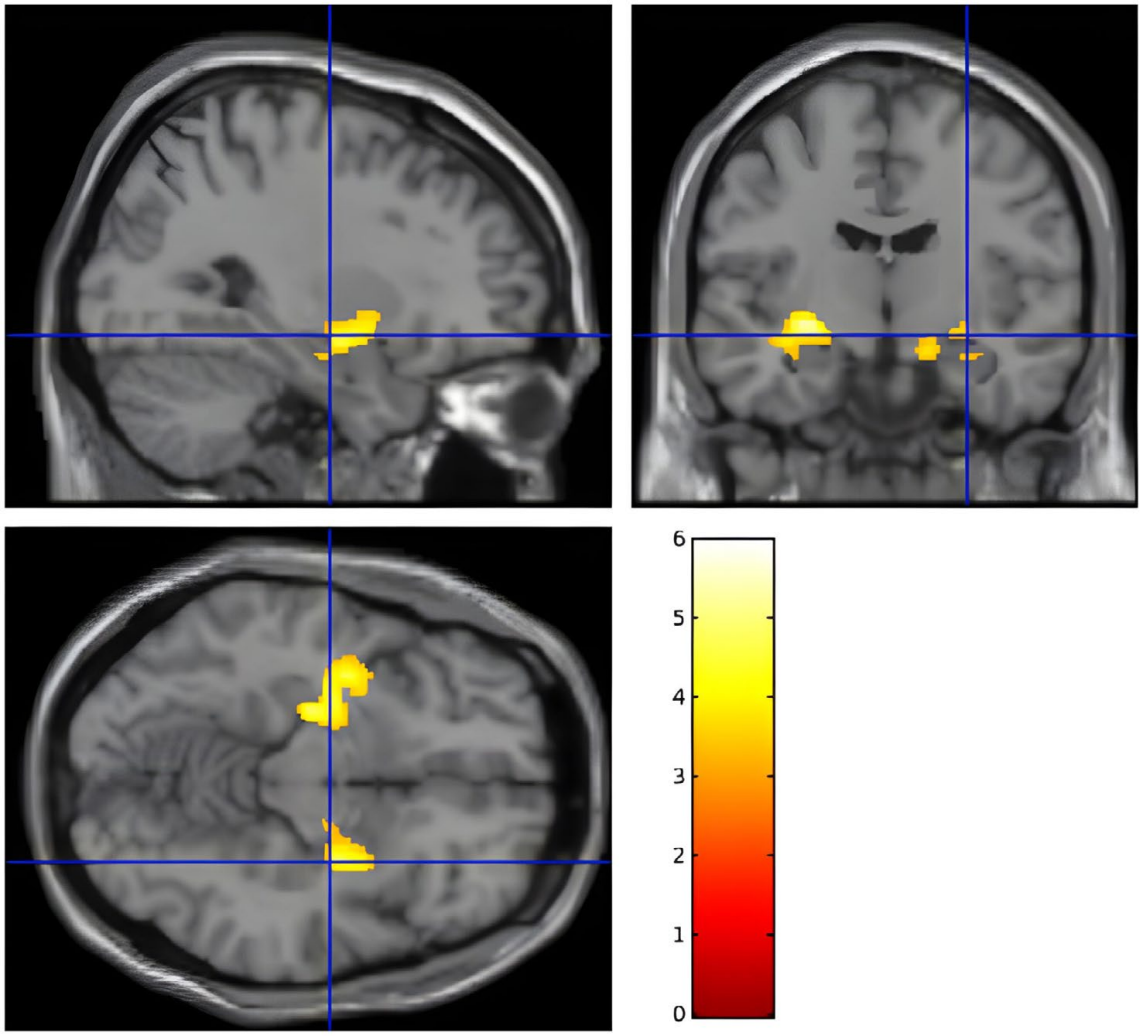
Voxel‐based morphometry analysis (CAT12) shows the bilateral amygdala and hippocampus volumes were significantly larger in the *APOE* ε4 carriers than in the noncarriers (FDR Corrected, *p* < .005, extent cluster threshold of 100 voxels).

VBM analysis revealed significantly larger bilateral hippocampal and amygdalar volumes in carriers compared with noncarriers (Figure 1). This finding was corroborated by atlas‐based volumetric analysis. In contrast, the atlas‐based analysis indicated a reduction in cortical thickness in carriers compared with noncarriers, particularly in the left lingual gyrus, right lateral occipital gyrus, and right posterior cingulate (*p* < .05; Table 3).

**TABLE 3:** FreeSurfer regional volumes/thicknesses comparisons^a^.

		Carriers 20	Noncarriers 45	*p*‐values
Volume	Left hippocampus	3948 ± 69	3760 ± 46	.028
	Right hippocampus	4101 ± 73	3883 ± 49	.016
	Left amygdala	1606 ± 36	1487 ± 24	.008
	Right amygdala	1796 ± 37	1676 ± 24	.009
Thickness	Left lingual gyrus	1.863 ± 0.020	1.926 ± 0.013	.012
	Right lateral occipital gyrus	2.001 ± 0.021	2.058 ± 0.014	.026
	Right posterior cingulate	2.329 ± 0.028	2.405 ± 0.018	.027

^a^ Volumes are reported in cubic millimeters (mm^3^) and cortical thicknesses in millimeters (mm).

### 3.3 DTI analysis

#### 3.3.1 TBSS analysis

**FIGURE 2:**
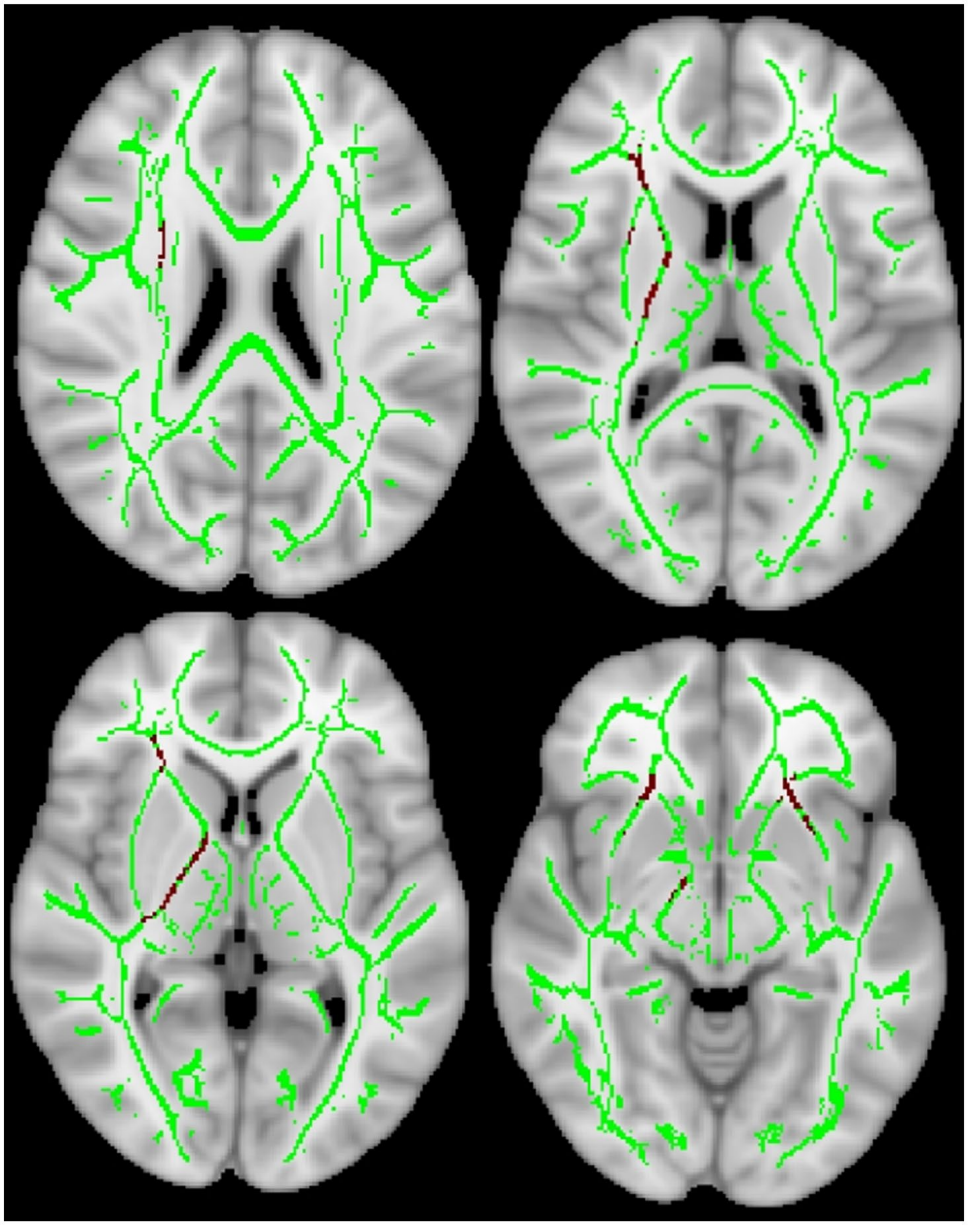
Tract‐based spatial statistics analysis shows a trend toward decreased fractional anisotropy (*p* < .10) in *APOE* ε4 carriers compared with the noncarriers, after controlling for disease duration. This trend was observed in the right internal capsule and anterior portion of the bilateral inferior fronto‐occipital fasciculus white matter tracts. Green represents the white matter skeleton, while red indicates regions showing a trend toward significant difference.

**FIGURE 3:**
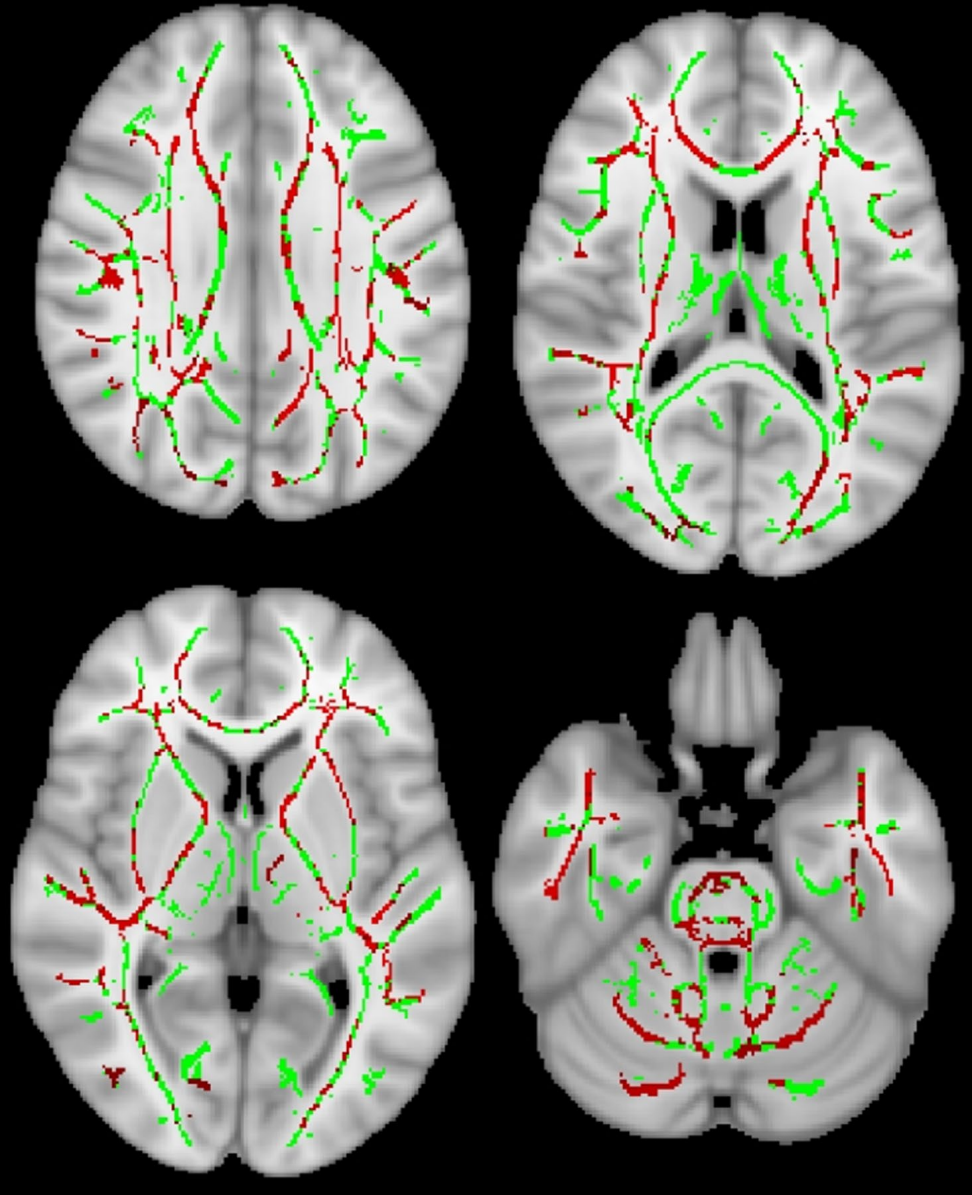
Tract‐Based Spatial Statistics analysis shows the mean diffusivity was significantly increased in the *APOE* ε4 carriers compared with the noncarriers in extensive white matter tracts. Green indicates the white matter tract skeleton, and red indicates the areas indicating significant difference.

TBSS revealed that compared with noncarriers, carriers tended to have decreased FA (*p* < .10) in the right internal capsule and anterior portion of the bilateral inferior fronto‐occipital fasciculus WM tracts (Figure 2), while MD was significantly increased across extensive WM tracts (Figure 3).

#### 3.3.2 DTI atlas‐based analysis

Compared with noncarriers, carriers exhibited significantly reduced FA values in the middle cerebellar peduncle and bilateral WM tracts, including the corticospinal tract, medial lemniscus, anterior and posterior limbs of the internal capsule, and inferior fronto‐occipital fasciculus. On the right side, FA reductions were observed in the anterior corona radiata, external capsule, and superior fronto‐occipital fasciculus, while on the left, they were noted in the superior and posterior corona radiata and the cingulum (cingulate gyrus) (Table S1).

MD values were significantly increased in carriers compared with noncarriers in the middle cerebellar peduncle, pontine crossing tract, and genu of the corpus callosum. Bilateral increases in MD were observed in the corticospinal tract, medial lemniscus, inferior cerebellar peduncle, internal capsule, corona radiata, sagittal stratum, external capsule, cingulum (hippocampus), fornix (cres)/stria terminalis, superior longitudinal fasciculus, and inferior fronto‐occipital fasciculus. MD increases were detected on the left side in the superior cerebellar peduncle, cingulum (cingulate gyrus), and uncinate fasciculus, while on the right side, changes were seen in the superior fronto‐occipital fasciculus and posterior thalamic radiation.

### 3.4 Correlation between volume/thickness and DTI findings

Only MD exhibited significant differences between carriers and noncarriers in both TBSS and atlas‐based analyses. Therefore, we further examined the correlation between MD values in WM tracts and the volumes/thicknesses of regions that exhibited significant group differences (Table 4). Hippocampal and amygdalar volumes were positively correlated with MD values across multiple WM tracts, except for the column and body of the fornix, where MD values showed a negative correlation. In contrast, the thickness of the left lingual gyrus consistently exhibited a negative correlation with MD values across multiple WM tracts.

**TABLE 4:** Correlation coefficients between regional volume/thickness and medial diffusivity (MD).

	L hippocampus	R hippocampus	L amygdala	R amygdala	L lingual thickness	R lateraloccipital thickness	R posteriorcingulate thickness
Pontine crossing tract	.223	.261	.231	.303^a^	−.004	.142	.141
Genu of corpus callosum	−.026	−.140	.171	.109	−.389^b^	−.245	−.094
Body of corpus callosum	.073	.002	.277^a^	.146	−.223	−.146	.055
Fornix (column and body)	−.366^b^	−.335^a^	−.189	−.039	−.213	−.134	−.212
Corticospinal tract R	.237	.200	.194	.268	.028	.093	.275^a^
Corticospinal tract L	.300^a^	.344^a^	.166	.189	.062	.007	.223
Medial lemniscus R	.275^a^	.235	.085	.312^a^	−.233	−.204	.071
Medial lemniscus L	.279^a^	.291^a^	.098	.244	−.125	−.136	.120
Inferior cerebellar peduncle R	.068	.018	−.050	.012	−.282^a^	−.163	−.023
Inferior cerebellar peduncle L	.007	.007	.009	.039	−.331^a^	−.254	−.020
Cerebral peduncle R	.145	.195	.278^a^	.276^a^	−.142	−.070	.082
Anterior limb of internal capsule R	.081	.071	.226	.070	−.279^a^	−.147	.115
Anterior limb of internal capsule L	.202	.125	.263	.252	−.338^a^	−.164	.124
Posterior limb of internal capsule R	−.005	.020	.292^a^	.141	−.306^a^	−.112	.019
Posterior limb of internal capsule L	.093	.028	.357^b^	.229	−.270	.031	−.027
Retrolenticular part of internal capsule R	.309^a^	.161	.307^a^	.195	−.103	−.021	.146
Anterior corona radiata R	.059	−.053	.155	.035	−.346^a^	−.250	.009
Anterior corona radiata L	.043	−.102	.117	.052	−.349^a^	−.151	.026
Superior corona radiata R	.056	−.020	.195	.091	−.367^b^	−.167	−.040
Superior corona radiata L	.013	−.072	.164	.082	−.428^b^	−.263	−.041
Posterior corona radiata R	.004	−.096	.104	.011	−.291^a^	−.166	−.078
Posterior corona radiata L	.004	−.102	.054	.008	−.335^a^	−.209	−.123
Sagittal stratum L	.140	.044	.305^a^	.220	−.263	−.107	−.057
External capsule R	.129	−.002	.339^a^	.177	−.310^a^	−.138	−.014
External capsule L	.166	.046	.281^a^	.245	−.338^a^	−.150	.042
Cingulum (cingulate gyrus) L	.065	.016	.253	.299^a^	−.216	−.185	.086
Cingulum (hippocampus) R	.299^a^	.232	.183	.222	−.143	−.069	.260
Fornix (cres) / Stria terminalis R	.309^a^	.279^a^	.427^b^	.295^a^	−.130	.013	.054
Superior longitudinal fasciculus R	.000	−.092	.125	.052	−.377^b^	−.237	−.115
Superior longitudinal fasciculus L	.013	−.094	.139	.079	−.403^b^	−.239	−.062
Inferior fronto‐occipital fasciculus R	.358^b^	.216	.377^b^	.266	−.164	−.056	.049
Inferior fronto‐occipital fasciculus L	.182	.035	.279^a^	.218	−.282^a^	−.083	.113

^a^ Correlation is significant at the 0.05 level (two‐tailed).

^b^ Correlation is significant at the 0.01 level (two‐tailed).

### 3.5 Correlation between volume/thickness and clinical scores

Hippocampus and Amygdala: Significant negative correlations with clinical scores (e.g., FT, AM, Negative Myoclonus, Negative Myoclonus Severity, Global Disability) suggest that larger volumes in these regions are associated with better clinical outcomes (Table 5).

**TABLE 5:** Correlation coefficients between regional volume/thickness and clinical scores.

	Left hippocampus	Right hippocampus	Left amygdala	Right amygdala	Left lingual gyrus	Right lateral occipital gyrus	Right posterior cingulate
UMRS							
SS	−.188	−.192	−.185	−.167	−.001	.007	−.035
FT	−.526**	−.544**	−.476**	−.413**	−.23	−.212	−.299*
AM	−.538**	−.557**	−.491**	−.426**	−.21	−.19	−.286*
Negative myoclonus	−.517**	−.531**	−.456**	−.395**	−.282*	−.266*	−.337**
Negative myoclonus severity	−.498**	−.510**	−.444**	−.389**	−.248	−.233	−.301*
Global disability	−.486**	−.517**	−.425**	−.344**	−.288*	−.267*	−.369**
QOLIE‐31							
Seizure worry	.106	.108	.109	.1	−.021	−.026	.001
Overall quality of life	.027	.054	.006	−.04	.107	.096	.147
Emotion	−.07	−.02	−.079	−.152	.05	.03	.13
Energy/fatigue	−.036	.009	−.042	−.11	.035	.016	.112
Cognitive function	.081	.102	.083	.047	−.014	−.027	.04
Medical effects	−.061	−.027	−.045	−.091	−.079	−.096	−.008
Social functioning	.145	.172	.121	.068	.113	.099	.169
Overall score	.056	.093	.048	−.013	.037	.019	.111
Health score	.04	.058	.039	.009	.003	−.007	.045

*Note*: ** indicates *p* < .01; * indicates <.05.

Abbreviations: FT, functional tests score; QOLIE‐1, the quality of life in epilepsy inventory‐31; SS, stimulus sensitivity score; UMRS, Unified Myoclonus Rating Scale.

Lingual Gyrus, Lateral Occipital Gyrus, and Posterior Cingulate Thickness: Negative correlations with clinical scores indicate that reduced cortical thickness in these regions is linked to worse clinical outcomes.

## 4 DISCUSSION

The current study matched patients with EPM1 by age, gender, and disease duration, revealing key findings about *APOE* ε4 carriers. These individuals exhibited reduced cortical thickness, which aligns with previous studies and is indicative of neurodegeneration. For instance,^29^ reported steeper age‐related declines in cortical microstructure among *APOE* ε4 carriers, particularly in the temporoparietal and frontal lobes. However, structural differences in young adults have been more heterogeneous, with studies showing subtle or inconsistent findings.^30^

In *APOE* ε4 carriers, more pronounced WM degeneration was evident in both motor and sensory tracts (e.g., corticospinal tract, medial lemniscus) as well as associative tracts (e.g., superior fronto‐occipital fasciculus, corona radiata). Decreased FA and increased MD in these tracts suggest reduced fiber coherence, demyelination, or axonal loss, indicative of structural degradation. This pattern is consistent with prior findings, including,^29^, ^31^ which reported significant microstructural deficits in *APOE* ε4 carriers.

An unexpected finding in EPM1 was the relatively preserved hippocampal and amygdalar volumes in *APOE* ε4 carriers, despite reduced cortical thickness. This contrasting pattern suggests that *APOE* ε4 may have distinct effects on cortical versus deep gray matter structures. Chronic cortical neuronal damage due to epileptic activity may contribute to cortical thinning, while the propagation of abnormal electrical activity might be less active in deeper structures, potentially limiting neuronal degeneration in subcortical regions such as the hippocampus and amygdala. This pattern contrasts with findings in Alzheimer's disease,^13^, ^22^ where *APOE* ε4 is strongly associated with accelerated hippocampal and amygdalar atrophy due to its role in promoting amyloid deposition, tau pathology, and neuroinflammation—pathological processes that directly affect these limbic structures. Unlike EPM1, Alzheimer's disease is characterized by intrinsic degenerative processes that prominently target the hippocampus. Thus, the preserved volumes observed in our EPM1 *APOE* ε4 carriers may reflect a disease‐specific interplay between epileptiform cortical damage and relative subcortical protection, offering a novel perspective on region‐dependent vulnerability in neurodegenerative and epileptic conditions.

*APOE* ε4 carriers also showed more severe WM degeneration, characterized by decreased FA and increased MD in WM tracts. The positive correlations between hippocampal/amygdalar volumes and MD values in WM tracts and the negative relationship between hippocampal/amygdalar volumes and UMRS scores suggest that preserved deep gray matter may mitigate the clinical impact of cortical thinning, underscoring its potential compensatory role in disease progression.

In earlier studies, larger hippocampal and amygdalar volumes have been shown to be associated with better emotional regulation, memory, and stress resilience, whereas a smaller hippocampus has been identified as a vulnerability factor for perceived stress.^32^ These structural advantages likely contribute to our finding that *APOE* ε4 carriers reported higher QOLIE‐31 scores in emotional well‐being, lower fatigue, and significantly improved perceptions of medical effects, despite comparable disease severity to noncarriers. The hippocampus plays a key role in stress adaptation, and the amygdala influences emotional processing, potentially supporting more effective emotional regulation and coping with medical challenges. We speculate that enhanced structural integrity in these regions may underlie greater psychological resilience, correlating with fewer adverse perceptions of medical effects and improved subjective well‐being. While only the domain of medical effects reached clear statistical significance, the consistent trend across multiple QOLIE‐31 subdomains—together with the negative correlations between limbic volumes and clinical severity—suggests the presence of a functionally meaningful phenotype within the EPM1 population. These results should, however, be interpreted with caution, as QOLIE‐31 is a self‐reported measure and therefore susceptible to personal and environmental influences. Although all participants were receiving standard clinical care for EPM1, individual differences in medication use, psychiatric comorbidities, psychosocial support, and rehabilitation engagement were not systematically assessed. These unmeasured variables may have contributed to the variability in quality‐of‐life outcomes and represent potential sources of residual confounding.

Limitations of our study include its cross‐sectional design, with assessments conducted at varying ages and disease stages, which introduces the risk of confounding interindividual variability with true temporal progression. This makes it difficult to draw firm conclusions about disease trajectories. Given the inherently progressive nature of EPM1, longitudinal studies are essential to confirm the observed findings, clarify their temporal dynamics, and better understand the evolution of structural and clinical changes over time. A potential survivor or severity bias must also be considered, as participation in advanced imaging studies generally requires a minimum level of clinical stability. This may have inadvertently excluded the most severely affected individuals, potentially including some *APOE* ε4 carriers. The relatively small sample size also limits generalizability. Nonetheless, our cohort included a broad range of clinical severity, with several patients requiring wheelchair assistance and demonstrating substantial variation in motor function and quality‐of‐life outcomes. Furthermore, as clinical severity and neuropsychological performance were assessed during a single study visit, this single‐time point measure may not reliably reflect disease severity or cognitive function because of the symptom fluctuations in EPM1. A more reliable and accurate method for assessing disease severity could be achieved through longitudinal monitoring or ecologically valid approaches, such as home‐based evaluation.^33^, ^34^ Without repeated neuropsychological testing, we were unable to assess whether the cortical thinning observed in *APOE* ε4 carriers corresponds to progressive cognitive decline. Future studies combining longitudinal imaging and serial neuropsychological evaluation will be critical for determining whether these structural changes reflect ongoing neurodegeneration with functional consequences.

The findings of this study underscore the importance of identifying biomarkers, such as *APOE*, to further uncover the mechanisms driving phenotypic variation in EPM1 patients. Like their established roles in other neurodegenerative disorders, such as Alzheimer's disease, these biomarkers could offer a more accessible approach to understanding the heterogeneity and progression of EPM1.

## 5 CONCLUSIONS

In conclusion, *APOE* ε4 carriers among EPM1 patients exhibited reduced cortical thickness and more severe WM degeneration, with decreased FA and increased MD in critical tracts. However, despite these structural changes, carriers showed larger hippocampal and amygdalar volumes, correlating with better emotional well‐being, lower fatigue, and improved medical effects. This study highlights the complex interplay between genetic factors and neurodegenerative processes, offering valuable insights into the progression of EPM1 and its impact on brain structure and function. Additionally, the potential role of biomarkers in uncovering mechanisms underlying phenotypic variability in EPM1 warrants further investigation.

## FUNDING INFORMATION

Saastamoinen Foundation. Folkhälsan Research Foundation.

## CONFLICT OF INTEREST STATEMENT

Reetta Kälviäinen: grants from the Academy of Finland, Saastamoinen Foundation, and Vaajasalo Foundation; honoraria from Eisai, Orion, Sandoz, Sanofi, and UCB; and honoraria for membership on the advisory boards of and consultation from Angelini Pharma, Eisai, Marinus Pharmaceuticals, Orion, Takeda, and UCB. Katri Silvennoinen: honoraria from Jazz Pharmaceuticals and UCB. All other authors have no relevant conflicts of interest to disclose.

**Test yourself**

1. Which of the following is a key characteristic of EPM1?

- Myoclonus and seizures
- Cognitive decline without motor symptoms
- Only seizures with no neurological symptoms

2. How does *APOE* ε4 carrier status affect the possibility of Alzheimer's disease?

- Protective role
- Increases risk
- Neutral effect

3. Which of the following structural changes were observed in EPM1 patients with *APOE* ε4?

- More pronounced white matter degeneration
- Smaller hippocampal and amygdalar volumes
- No changes in brain structure

*Answers may be found in the* *supporting information*

## Supporting information


Table S1.

Data S1.

## Data Availability

The data that support the findings of this study are available on request from the corresponding author. The data are not publicly available due to privacy or ethical restrictions.
