# Supplementary material for: Exploring the role of apolipoprotein ε4 in progressive myoclonic epilepsy type 1
Source: Epileptic Disord. 2025 Oct 3;28(1):55–67. doi: 10.1002/epd2.70112 (PMC12964178; doi:10.1002/epd2.70112)
Supplement: Supplementary file 1 — Table S1. [file EPD2-28-55-s001.docx]

| Table S1. Fractional anisotropy (FA) and medial diffusivity (MD) changes in atlas-based analysis and potential effect on brain functions | | | |
| --- | --- | --- | --- |
| White Matter Tract | FA Findings (Carriers vs. Non-Carriers) | MD Findings (Carriers vs. Non-Carriers) | Interpretation |
| Middle cerebellar peduncle | ↓ FA in carriers | ↑ MD in carriers | Indicates structural degradation, likely due to demyelination or axonal damage, impacting motor coordination. |
| Bilateral corticospinal tract | ↓ FA in carriers | ↑ MD in carriers | Suggests reduced integrity of motor pathways, consistent with potential motor control issues. |
| Medial lemniscus | ↓ FA in carriers | ↑ MD in carriers | Points to potential sensory processing deficits due to structural compromise in sensory pathways. |
| Anterior and posterior limbs of internal capsule | ↓ FA in carriers | ↑ MD in carriers | Suggests disruptions in sensorimotor integration, possibly impacting motor and sensory functions. |
| Inferior fronto-occipital fasciculus | ↓ FA in carriers | ↑ MD in carriers | Indicates structural degradation, potentially impacting visual processing and cognitive integration across hemispheres. |
| Right anterior corona radiata | ↓ FA in carriers | ↑ MD in carriers | Reduced FA with increased MD suggests loss of fiber integrity, which may impact cognitive and emotional regulation functions associated with frontal brain regions. |
| Right external capsule | ↓ FA in carriers | ↑ MD in carriers | Implies compromised structural integrity, potentially impacting communication between cortical and subcortical regions. |
| Right superior fronto-occipital fasciculus | ↓ FA in carriers | ↑ MD in carriers | Suggests reduced connectivity in pathways supporting visual and spatial processing. |
| Left superior corona radiata | ↓ FA in carriers | ↑ MD in carriers | Indicates potential motor and sensory processing deficits due to structural degradation. |
| Left posterior corona radiata | ↓ FA in carriers | ↑ MD in carriers | Suggests potential sensory processing or visuospatial deficits. |
| Left cingulum (cingulate gyrus) | ↓ FA in carriers | ↑ MD in carriers | Points to possible cognitive control and memory function impairment due to compromised cingulum integrity. |
| Pontine crossing tract | — | ↑ MD in carriers | Increased MD suggests structural degradation, potentially affecting coordination and integration of motor signals. |
| Genu of corpus callosum | — | ↑ MD in carriers | Elevated MD suggests disrupted connectivity between hemispheres, possibly affecting interhemispheric communication. |
| Cingulum (hippocampus) | — | ↑ MD in carriers | Increased MD indicates potential degradation impacting memory processes and limbic connectivity. |
| Fornix (cres) / Stria terminalis | — | ↑ MD in carriers | Higher MD suggests structural degradation that may impact memory and limbic system connectivity. |
| Superior longitudinal fasciculus | — | ↑ MD in carriers | Increased MD suggests microstructural degradation, potentially affecting long-range cognitive connectivity. |
| Superior cerebellar peduncle (Left) | — | ↑ MD in carriers | Elevated MD indicates structural degradation, potentially impacting motor coordination. |
| Uncinate fasciculus (Left) | — | ↑ MD in carriers | Higher MD suggests compromised structural integrity in tracts related to emotion and memory. |
| Right posterior thalamic radiation | — | ↑ MD in carriers | Increased MD suggests structural compromise in sensory integration pathways. |
