# Supplementary material for: Exploring the role of apolipoprotein ε4 in progressive myoclonic epilepsy type 1
Source: Epileptic Disord. 2025 Oct 3;28(1):55–67. doi: 10.1002/epd2.70112 (PMC12964178; doi:10.1002/epd2.70112)
Supplement: Supplementary file 2 — Data S1. [file EPD2-28-55-s002.docx]

Answer to question 1: (a) Myoclonus and seizures.

Answer to question 2: (b) Increases risk.

Answer to question 3: (a) More pronounced white matter degeneration.
